# Characterization of meningiomas with synthetic imaging

**DOI:** 10.1002/brb3.2769

**Published:** 2022-10-12

**Authors:** Riccardo Ludovichetti, Bénédicte Delattre, José Boto, Daniela LaGrange, Torstein Meling, Maria Isabel Vargas

**Affiliations:** ^1^ Division of Radiology Diagnostic Department, Geneva University Hospitals Geneva Switzerland; ^2^ Division of Neuroradiology University Hospitals of Zurich; ^3^ Division of Neuroradiology Diagnostic Department, Geneva University Hospitals Geneva Switzerland; ^4^ Division of Neurosurgery Neurosciences Department, Geneva University Hospitals Geneva Switzerland; ^5^ Faculty of Médecine University of Geneva Geneva Switzerland

**Keywords:** biomarkers, meningiomas, MRI, relaxivity values, synthetic imaging

## Abstract

**Introduction:**

Synthetic magnetic resonance imaging (SyMRI) is a novel quantitative and qualitative technique that permits the reconstruction of multiple image contrasts and quantitative maps from a single scan, thereby providing quantitative information and reducing scan times. The purpose of this study is to characterize intracranial meningiomas using SyMRI.

**Methods:**

The study included 35 patients with meningiomas (6 males, 29 females; mean age 61 ± 17 years; range 21–90 years). Using 3T MR scanners, SyMRI was performed in addition to conventional FSET2, FLAIR, DWI, T1, and T1 with gadolinium. SyMRI software was used to generate T1, T2, and PD quantitative maps. Osirix MD was used to measure quantitative values of T1, T2, and PD using a ROI.

**Results:**

We analyzed 42 meningiomas, 8 of which were associated with edema, and 5 contained calcifications.

Mean relaxivity values of meningiomas on synthetic T1, T2, and PD maps at 3T MRI were 1382.6 ± 391.7 ms, 95.6 ± 36.5 ms, and 89.1 ± 9.7 pu, respectively. Signal intensities in terms of T1, T2, and PD did not differ significantly between meningiomas with and without edema (*p* = .994, *p* = .356, and *p* = .221, respectively), nor between meningiomas containing and not containing calcifications (*p* = .840, *p* = .710, and *p* = .455, respectively). Values of T1 and T2 measured in meningiomas and the normal‐appearing white matter approximated reference values found in the literature with other quantitative methods.

**Conclusion:**

The presented method offers a novel approach to characterize meningiomas through their relaxation parameters measured with a SyMRI sequence.

## Graphical abstract

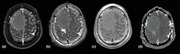

## 1 INTRODUCTION

Magnetic resonance imaging (MRI) is widely accepted as the modality of choice for diagnostic imaging of the brain due to its excellent display of soft‐tissue contrast. In conventional MRI, images are generated by sequential data acquisition with specific scanner settings and the process requires a relatively long acquisition time (Lee et al., 2017). In contrast, synthetic MRI (SyMRI) is a novel imaging technique that allows multiple contrast‐weighted images to be generated based on measurements of tissue relaxivity in a single acquisition using a multiecho, multidelay saturation recovery spin‐echo sequence (Gonçalves et al., 2018). This offers the possibility of shortening the acquisition duration (Vargas et al., 2018) and allows the determination of tissue properties in terms of T1, T2, and PD.

Several studies have been conducted to validate the feasibility of SyMRI of the brain in adults and children (Betts et al., 2016; Blystad et al., 2012; Hagiwara et al., 2017) but to our knowledge, it has not yet been used for the characterization of meningiomas. In view of this, the purpose of this study is to characterize meningiomas using SyMRI.

## 2 MATERIALS AND METHODS

### 2.1 Patients

The local ethics committee on research involving humans approved this retrospective study (protocol blinded for anonymity) and informed consent was waived. A synthetic MRI was added to our usual meningioma imaging protocol in 35 patients (6 males, 29 females; mean age of 61 ± 17 years; age range 21–90 years). The patients were referred to our institution with a clinical indication for neuroimaging in the follow‐up of a previously diagnosed meningioma between October 2019 and October 2020. All patients had known meningiomas of a major axis >8 mm, to minimize partial volume effects. Patients with smaller meningiomas were not included in this study. Eleven patients underwent neurosurgery after the MRI, with histopathological confirmation of the diagnosis, and 24 patients had lesions with radiologic features and follow‐up on MRI congruent with meningiomas. All patients were 18 years old or older and had no contraindication to MR imaging.

#### 2.1.1 Image acquisition

Two sets of images were created for each patient using conventional and synthetic sequences. Synthetic MRI was performed on two different 3T MR scanners: Siemens Skyra 3.0T (Siemens Healthcare, Erlangen, Germany) and Philips Ellition X 3.0T (Philips Healthcare, Best, the Netherlands) in addition to the conventional sequences FSET2, FLAIR, DWI, T1, and T1 with gadolinium.

The QRAPMASTER sequence (quantification of relaxation times and proton density by the multiecho acquisition of saturation recovery with turbo‐spin‐echo readout) on the Siemens scanner and the MDME sequence (multiple‐dynamic multiple‐echo) on the Philips scanner were performed to retrieve the T1 and T2 relaxation times as well as the PD. The QRAPMASTER and the MDME sequence are multispin‐echo saturation‐recovery sequences, with 4 different saturation delays and 2 echoes. On the Siemens scanner, the technical parameters were: in‐plane resolution 0.7 × 0.7 mm^2^, TE 23 and 100 ms, TR 4790 ms, slice thickness 4 mm with 0.4 mm gap, acquisition time 5 min 27 s for 33 slices. On the Philips scanner, the parameters were: in‐plane resolution 0.5 × 0.5 mm^2^, TE 13 and 100 ms, TR 4517 ms, slice thickness 4 mm with 1 mm gap, acquisition time 6 min 10 s for 30 slices.

**FIGURE 1:**
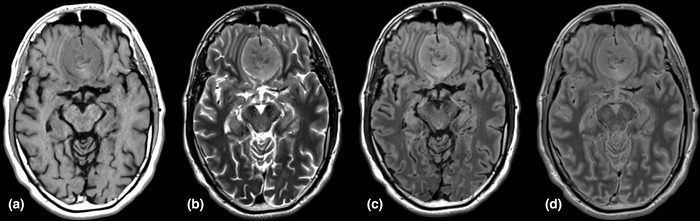
SyMRI acquired T1‐weighted (a), T2‐weighted (b), T2‐FLAIR‐weighted (c), and PD‐weighted (d) images on a parasagittal meningioma, reconstructed using the SyMRI software, Version 11.2 Ext. 2 (Synthetic MR AB, Linköping, Sweden)

QRAPMASTER and MDME data were reconstructed outside the clinical care environment with the SyMRI software, version 11.2 Ext. 2 (Synthetic MR AB, Linköping, Sweden) to generate T1‐, T2‐, T2‐FLAIR‐, and PD‐weighted images (Figure 1) and T1, T2, and PD quantitative maps. OsiriX MD (Pixmeo, Bernex, Switzerland) DICOM viewer was then used to measure quantitative values of T1, T2, and PD of the meningiomas and normal‐appearing white matter using an ROI. ADC mean values were also calculated on parametric maps. ROIs were placed by a radiology resident with 4 years of experience in radiology and were checked by a neuroradiologist with 15 years of experience in diagnostic neuroradiology.

**FIGURE 2:**
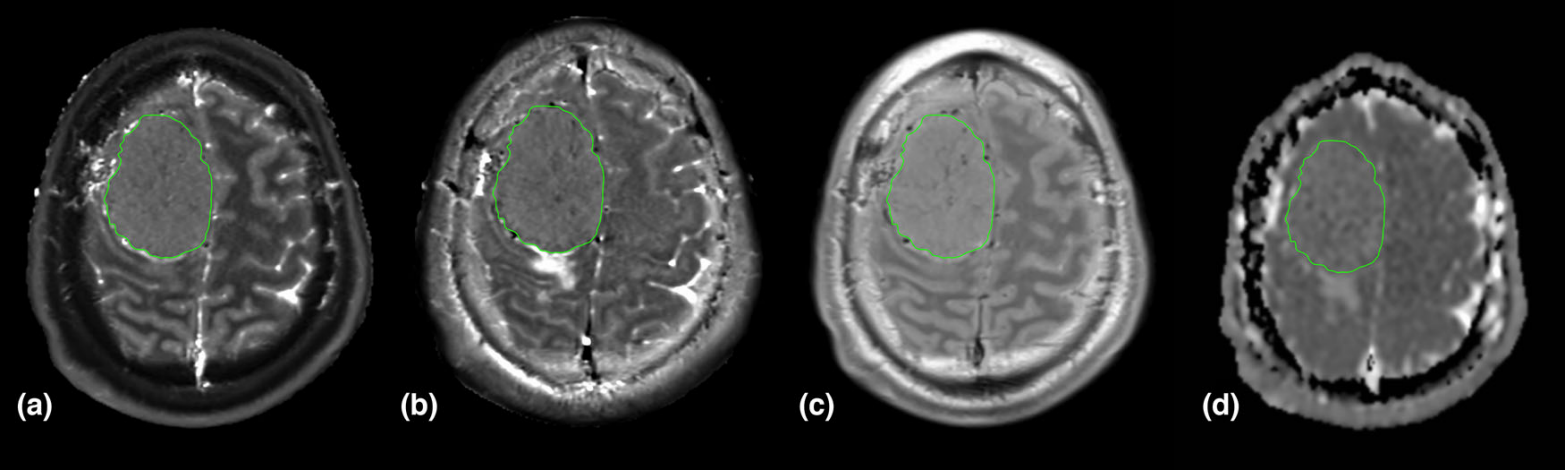
ROI placed on a frontal right parasagittal meningioma on SyMRI acquired T1 (a), T2 (b), and PD (c) quantitative maps and on the ADC map (d) to measure quantitative values

**FIGURE 3:**
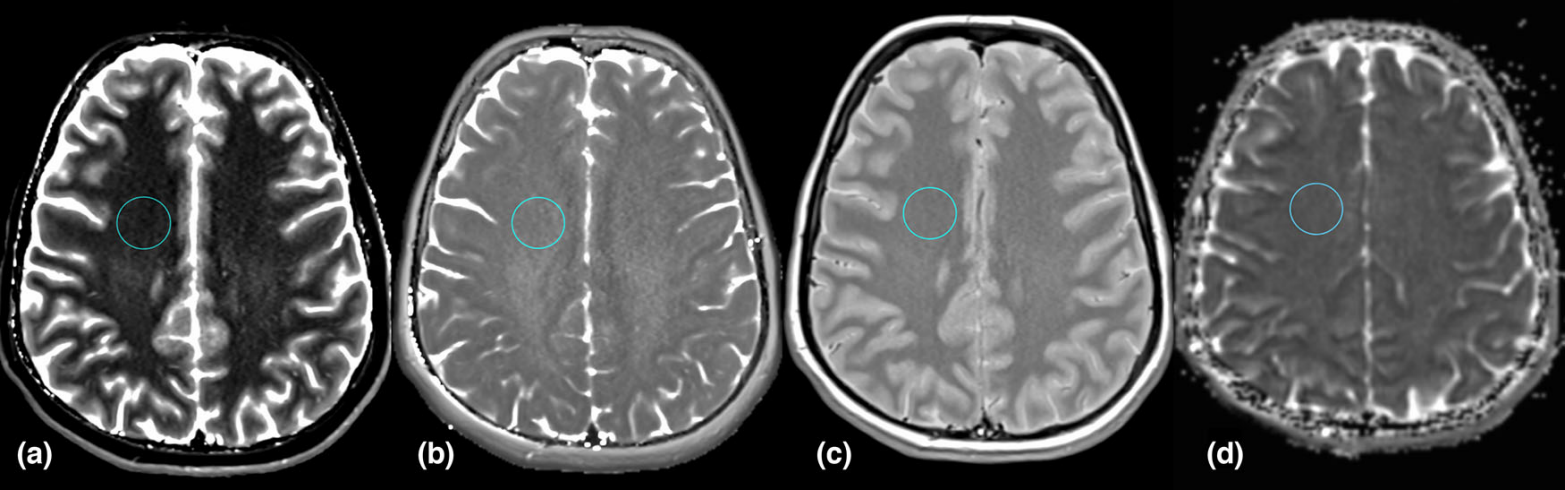
ROI placed in the normal appearing white matter of the centrum semiovale, on the contralateral side of the lesion, on SyMRI acquired T1 (a), T2 (b), and PD (c) quantitative maps and on the ADC map (d)

A single ROI was placed on a single axial slice on T1, T2, PD, and ADC quantitative maps to include the maximum possible area of the meningioma avoiding partial volume effects but so as not to include normal adjacent structures and perilesional edema (Figure 2). Calcifications were always included in the ROIs when present. No meningioma had necrosis or cystic regions. For each patient, a circular ROI covering an area of 3.25 cm^2^ was placed in the normal‐appearing white matter of the centrum semiovale on the contralateral side of the lesion or of the majority of lesions (Figure 3).

### 2.2 Statistical analysis

Descriptive statistics were obtained for the means of T1, T2, PD, and ADC values, including the skewness and kurtosis of the distributions by group to be compared (males vs. females, meningiomas vs. normal white matter, calcified vs. noncalcified meningiomas, meningiomas associated vs. not associated with edema). In order to determine whether the distributions were excessively nonnormal for the purposes of conducting parametric tests, we used the criteria of skewness between −2 and 2 and kurtosis between −9 and 9 (Schmider et al., 2010).

If data were not excessively nonnormally distributed, the means of the relaxation values were compared between groups by independent samples *T* test. Equality of variances was assessed by Levene's test, which if significant led to equal variances not being assumed.

If data were excessively nonnormally distributed, the nonparametric Mann–Whitney *U* test was used to compare medians between groups.

Liner relationships between continuous variables were evaluated by Pearson's correlation.

The statistical analysis was conducted on SPSS (version 22) and a .05 two‐tailed significance level was used for all statistical tests.

## 3 RESULTS

A total of 42 meningiomas found in 35 patients were included in this study (1 patient had 5 meningiomas, 3 patients had 2 meningiomas, and 31 patients had 1 meningioma). Clinical indications for MR imaging were presurgical evaluation and tumor follow‐up. The mean and standard deviation of the major axis of meningiomas were 33.52 ± 23.7 mm (maximum size: 130 mm, minimum size: 9 mm, median size: 28.5 mm).

The mean relaxation times of all meningiomas in Synthetic T1, T2, and the values of PD at 3T MRI were 1382.6 ± 391.7 ms, 95.6 ± 36.5 ms, and 89.1 ± 9.7 pu, respectively. The ADC mean value was 0.96 ± 0.25 × 10^–3^ mm^2^/s. The relaxation times of Synthetic T1, T2, and PD did not differ significantly between males and females (*p* = .874, *p* = .234, *p* = .453, respectively). A significant positive correlation (*p* < .01) was found between the mean values of T2 and ADC (*r* = 0.728).

Eight meningiomas had perilesional edema. The mean values of T1, T2, PD, and ADC of meningiomas with perilesional edema were 1381.7 ± 105 ms, 84.7 ± 11.13 ms, 85.3 ± 3.19 pu, and 0.81 ± 0.061 × 10^−3^ mm^2^/s, respectively. The relaxation times on Synthetic T1, T2, and PD of meningiomas with and without edema did not differ significantly (*p* = .994, *p* = .356, and *p* = .221, respectively). A statistically significant difference (*p* < .01) was found between the mean ADC values of meningiomas with or without perilesional edema; those with perilesional edema had a lower ADC value.

**FIGURE 4:**
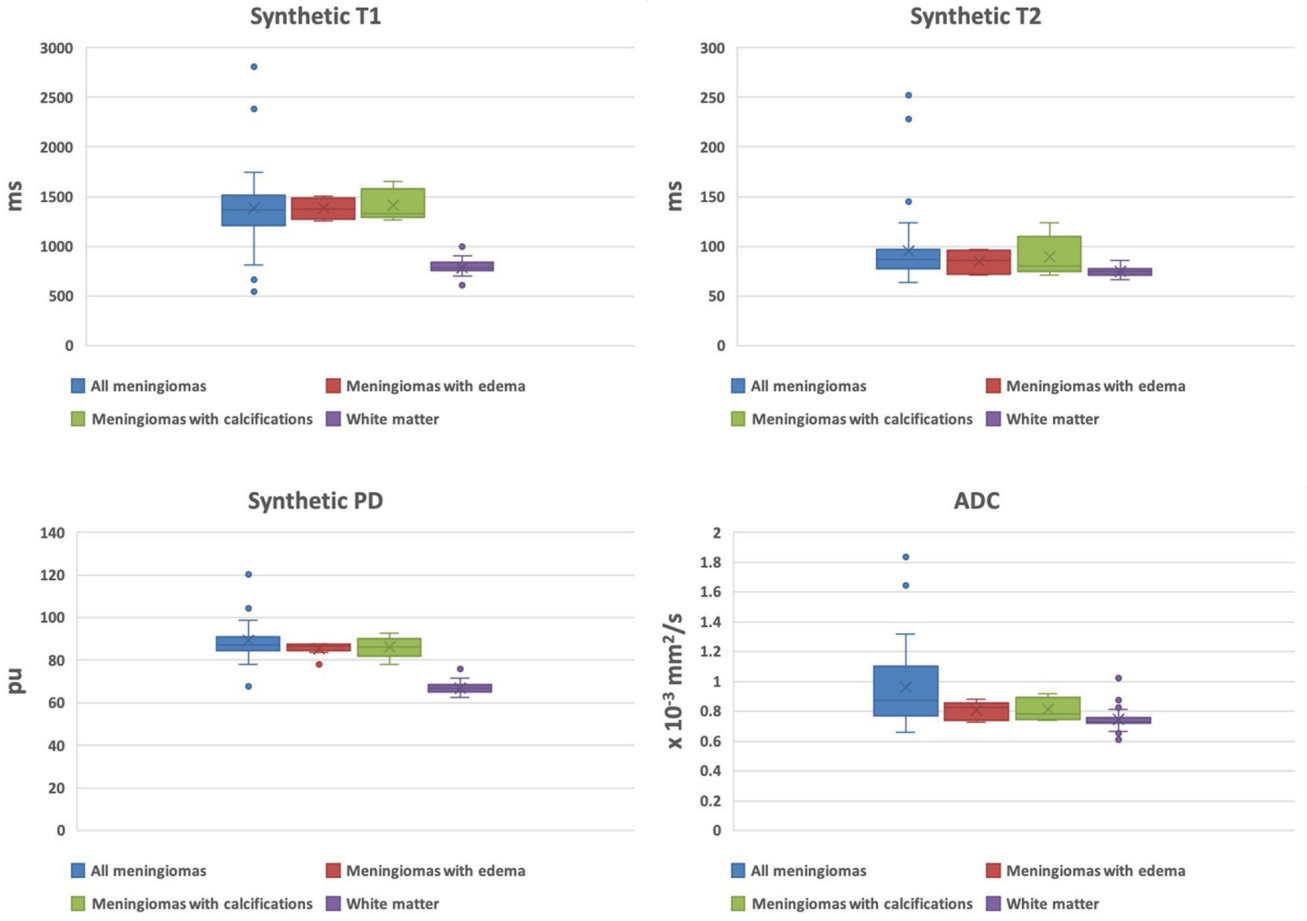
Box‐plots of synthetic T1, T2, PD, and ADC values of meningiomas (considered together and associated with edema and calcifications) and white matter

Five meningiomas contained calcifications. The mean values of T1, T2, PD, and ADC of meningiomas containing calcifications were 1416.40 ± 161.42 ms, 89.76 ± 21.04 ms, 86.05 ± 5.25 pu, and 0.81 ± 0.08 × 10^−3^ mm^2^/s, respectively. The relaxation times on Synthetic T1, T2, and PD of meningiomas with and without calcifications did not differ significantly (*p* = .840, *p* = .710, and *p* = .455, respectively). No significant difference (*p* = .155) was found between ADC values of meningiomas containing or not containing calcifications. Results are resumed in Table 1 and Figure 4.

**TABLE 1:** Quantitative values (synthetic acquired for T1, T2, and PD) in meningiomas ±edema and ±calcifications

	Edema			Calcifications		
	Yes	No	*p* Value	Yes	No	*p* Value
T1 (ms)	1381.72 ± 105.02	1382.82 ± 433.96	.994	1416.40 ± 161.42	1378.04 ± 414.37	.84
T2 (ms)	84.7 ± 11.13	98.13 ± 39.93	.356	89.76 ± 21.04	96.36 ± 38.26	.71
PD (pu)	85.32 ± 3.19	90.04 ± 10.52	.221	86.05 ± 5.26	89.56 ± 10.14	.455
ADC (10^−3^ mm^2^/s)	0.81 ± 0.06	1 ± 0.27	<.001	0.81 ± 0.08	0.98 ± 0.26	.155

*Note*: Values shown as mean ± standard deviation.

Concerning normal‐appearing white matter, the mean values of the relaxation times on synthetic T1, T2, and PD at 3T MRI were 801.14 ± 68.4 ms, 74.28 ± 4.5 ms, and 67.44 ± 2.6 pu, respectively. The mean ADC value of normal‐appearing white matter was 0.75 ± 0.07 × 10^–3^ mm^2^/s. A statistically significant difference (*p* < .01) was found between the means of all values of meningiomas and normal‐appearing white matter.

## 4 DISCUSSION

The clinical use of synthetic MRI (SyMRI) in neuroradiology has already been established. Several studies have focused on the diagnostic performance of this technique and on the possibility of producing images comparable with those of conventional sequences in a clinical setting (Blystad et al., 2012, Tanenbaum et al., 2017), as well as across multiple neurologic conditions, such as multiple sclerosis (Granberg et al., 2016), tumors (Blystad et al., 2020), and in pediatric neuroradiology (Betts et al., 2016; Lee et al., 2017; West et al., 2017). A recent use of SyMRI was for whole‐brain segmentation and volume estimation (Serai et al., 2019). A review by Hagiwara et al. (2017) focused on the use of SyMRI on the brain to find a specific quantification method and synthesis of contrast‐weighted images based on the acquired absolute values, as well as on the automatic segmentation of brain tissue. The automatic brain segmentation, along with the measurements of myelin, showed promising results in neurodegenerative brain diseases and tumoral diseases. Moreover, a recent study focused on the feasibility of synthetic MRI for spine imaging and to determine the normal values of T1, T2, and PD for the spine and spinal cord (Drake‐Pérez et al., 2018; Vargas et al., 2018).

Currently, the normal method used by neuroradiologists when interpreting MRI scans consists of comparing different tissues with different signals in the same slice. However, absolute signal intensity on standard MR images has no direct meaning, since it depends on several acquisition parameters as well as on the intrinsic properties of each type of scanner. An absolute scale, and one that could allow an objective characterization of diseased tissues while enabling a comparison between those tissues and normal brain in a repeatable manner, is provided by the absolute quantification of tissue parameters, such as T1, T2, and PD. SyMRI‐acquired relaxation values are reproducible, do not depend on the MR scanner, and are free from variations in the pulse sequence (Hagiwara et al., 2017). Absolute quantification of inverse relaxation rates (R1 and R2) and proton density (PD) has already been reported to allow characterization of brain disease (West et al., 2014). A recent study used SyMRI in malignant gliomas to acquire quantitative values to investigate the presence of contrast enhancement in peritumoral edema, concluding that relaxometry can detect areas of nonvisible contrast enhancement (Blystad et al., 2020).

The use of T1 relaxation times has also been used in the therapeutic monitoring of head and neck tumors (Wagner‐Manslau et al., 1994). Moreover, a recent study by Zhang et al. (2018) investigated the relationship between MRI features and pathological parameters in WHO I grade meningiomas, concluding that T1 and T2 signals can help predict pathological meningioma WHO I subtype.

The quantitative analysis of SyMRI generated maps along with the visual analysis of SyMRI generated weighted imaged could also help determine meningiomas subtypes, but further studies are needed to confirm this relationship.

A study by Kang et al. (2018) has recently applied synthetic MRI in patients with brain metastasis to investigate the time‐dependent effects of contrast medium on longitudinal relaxation rate (R1) and transverse relaxation rate (R2), concluding that relaxation rates were larger after contrast injection. Another study from Hagiwara et al. (2016) has examined the use of contrast‐enhanced synthetic MRI for detecting brain metastases, showing promising results.

The scan time for synthetic MRI is longer than that of a conventional postcontrast T1, but the possibility to generate multiple image contrasts outside the clinical environment after one single acquisition, therefore avoiding multiple scans, is a clear advantage of SyMRI (Hagiwara et al., 2016). Given these preliminary results, the use of both pre‐ and postcontrast SyMRI could be useful to identify pathological patterns in brain pathology and to optimize the current imaging protocols.

To our knowledge, this is the first study exploring the potential utility of SyMRI for the characterization of meningiomas. Meningiomas are the most common nonglial primary intracranial tumors (Rockhill et al., 2007), arising from arachnoid meningothelial cells. They are extra‐axial tumors, with a specific appearance on standard MRI acquisition, typically T1 isointense to slightly hypointense and T2 hyperintense to the cortex. While routinely acquired T1, T2, and postcontrast T1 are usually sufficient to make the diagnosis (Tamrazi et al., 2016) and SyMRI generated T1 and T2 weighted images were sufficient to replace the routinely acquired T1 and T2 in this population, the interest of this study is to acquire disease‐specific absolute quantitative values with the use of SyMRI.

To investigate the consistency of our technique, we compared our relaxation times of T1 and T2 for the normal‐appearing white matter to values found in the literature with other quantitative methods and these values were very similar. Wansapura et al. (1999) obtained the following T1 and T2 values for normal‐appearing white matter using saturation recovery and multiple spin‐echo imaging methods at 3 Tesla: T1 = 832 ± 10 ms, T2 = 79.6 ± 0.6 ms. These were similar to our mean values of T1 (801.14 ± 68.4 ms) and T2 (74.28 ± 4.5 ms) using the SyMRI. Another study by Parry et al. (2002) used a T1 mapping technique at 3T in healthy subjects, obtaining a T1 mean value of 891 ± 23 ms, in the same range as our values.

We also compared our relaxation times of T1 and T2 for meningiomas obtained with SyMRI to those found in the literature with other quantitative methods. A study by Komiyama et al. (1987) investigated 12 patients with meningiomas using a 0.5T MR, obtaining values for T1 (805.92 ± 111.3 ms) and T2 (78.24 ± 14.45 ms). In this study, T1 and T2 values in normal‐appearing white matter were measured in the frontal lobe, occipital lobe, and internal capsule, the highest values found being in the internal capsule with T1 and T2 relaxation times of 478.2 ± 72.9 ms and 75.4 ± 4.9 ms, respectively. The results from this study are similar to our results for T2 values and lower than ours for T1 values both in meningiomas and in normal‐appearing white matter. This is because T1 relaxation times depend on the strength of the magnetic field, as demonstrated in a study by Stanisz et al. (2005) in particular T1 relaxation time is lower if the magnetic field is lower. In the same study, T2 relaxation times have proven to be independent of magnetic field differences, which is coherent to our results. A recent study by Yamada et al. (2021) explored the potential of the T2 value for assessment of meningioma consistency using a 3T MRI scanner on 18 meningiomas. The mean and the standard deviation of T2 relaxation time were, respectively, 104.27 ms and 87.45 ms, in the same range as our results.

The apparent diffusion coefficient (ADC) measures the magnitude of diffusion of water molecules in tissues and can be quantified by analyzing diffusion‐weighted imaging (DWI). We calculated ADC values for all meningiomas and normal‐appearing white matter because we considered ADC to be part of the quantitative description of meningiomas. We also intended to investigate whether ADC variation could correlate with other parameters. ADC mean values for meningiomas were similar to those found in the literature (Meyer et al., 2020). A study by Meyer et al. (2020) showed a mean value for benign meningiomas on 3T MR of 0.94 × 10^–3^ mm^2^/s, which was similar to our mean value of 0.96 × 10^−3^ mm^2^/s.

**FIGURE 5:**
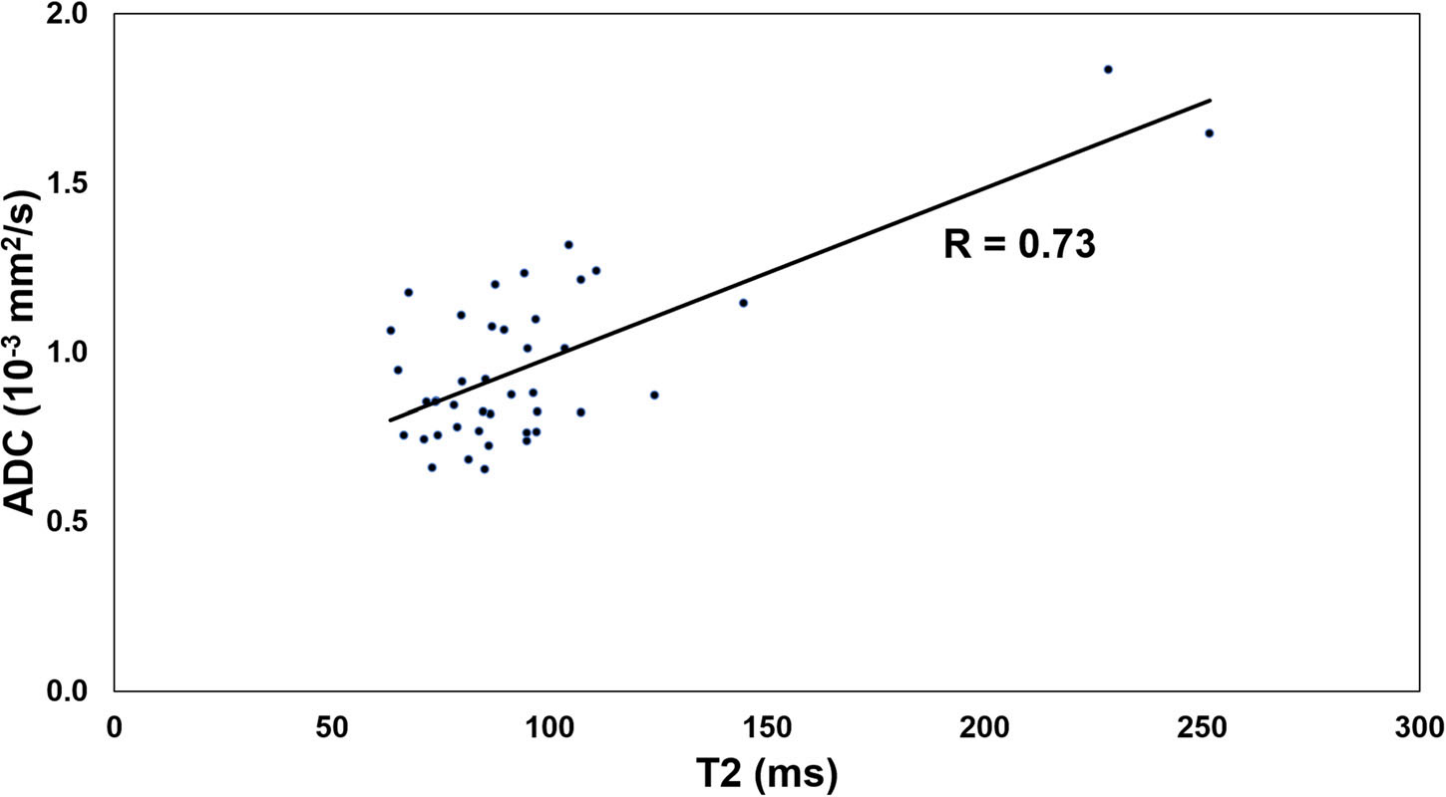
Correlation between mean values of T2 and ADC for meningiomas

We found a significant positive correlation (*p* < .01) between mean values of T2 and ADC (*r* = 0.728) for meningiomas (Figure 5). Similarly, Oh et al. (2005) found a strong correlation between ADC and T2 in meningiomas and metastases, as well as in gliomas. A study from Sugahara et al. (1999) found a correlation between tumor cellularity and ADC values for gliomas. Lower T2 values might be associated with lower water content, and therefore higher cellularity, as hypothesized by Oh et al. (2005). Despite this correlation, we found that the distribution of T2 values was more homogeneous than that of ADC values and therefore, in our opinion, more representative of meningiomas. As expected, all parameters investigated (T1, T2, PD, and ADC) were significantly different between meningiomas and normal‐appearing white matter.

We investigated the visual homogeneity of quantitative values for T1, T2, PD, and ADC for the normal‐appearing white matter to investigate their distribution when acquired through synthetic MRI. All results were very homogeneous. Results for meningiomas were visually homogeneous for T2 and PD, and less homogeneous for T1 and ADC (Figure 4).

There were some outliers in our data that could not be accounted for by unusual findings on the conventional weighted images, gender differences, the presence of edema or calcifications in meningiomas, meningioma size, or the presence of artifacts in any of the parameters. These outliers might therefore be due to an intrinsic heterogeneity in the composition of meningiomas, with T1 and ADC being the most sensitive parameters. Alternatively, they might be due to factors that we did not take into account in our study. Nevertheless, we believe that, despite the presence of these outliers, our results can be a useful tool to characterize meningiomas and serve as a basis for further investigation.

No significant difference in T1, T2, and PD values were found between our subgroups, in particular between meningiomas containing or not containing calcifications, or between meningiomas associated or not with edema, suggesting that these characteristics do not impact quantitative relaxation times. ADC values were significantly lower in meningiomas associated with perilesional edema. Presumably, this reflects changes caused by swelling of the surrounding tissue, causing restriction of diffusion.

Our study has some limitations, one of these being the relatively small sample size. Further investigation with a larger number of participants, focusing on more types of brain tumors, could provide objective quantitative values related to tissue properties in a disease‐specific manner. Another limitation is the fact that we could not find a plausible explanation for the outlier values of T1, T2, and PD through the factors that we investigated. A third limitation relates to the shortage of previous studies using quantitative methods to describe meningiomas as well as no studies using a 3T scanner, making it therefore difficult to find values for comparison.

## 5 CONCLUSION

In conclusion, Synthetic MR brain imaging offers a novel approach to the characterization of meningiomas by the addition of relaxation parameters to the imaging workup of these lesions in a short acquisition time. We believe that this approach will gain a larger scope in the future for the analysis of brain tumors, with an impact on treatment and follow‐up, and could replace some of the currently used standard imaging protocols.

## CONFLICT OF INTEREST

The authors have no relevant financial or nonfinancial interests to disclose.

### ETHICAL APPROVAL

The local ethics committee on research involving humans (Commission Cantonale d'Ethique de la Recherche sur l’être humain‐ CCER) approved this study. This study was performed in accordance with the ethical standards as laid down in the 1964 Declaration of Helsinki and its later amendments.

### PATIENT CONSENT

Patient informed consent (written and oral) was waived from the local ethics committee on research involving humans.

### PERMISSION TO REPRODUCE MATERIAL FROM OTHER SOURCES

No copyrighted works owned by third parties are included in this work.

### PEER REVIEW

The peer review history for this article is available at https://publons.com/publon/10.1002/brb3.2769

## Data Availability

The data that support the findings of this study are available from the corresponding author upon reasonable request.
